# The comparison of dyslipidemia and serum uric acid in patients with gout and asymptomatic hyperuricemia: a cross-sectional study

**DOI:** 10.1186/s12944-020-1197-y

**Published:** 2020-03-03

**Authors:** Jing Liang, Yutong Jiang, Yefei Huang, Wei Song, Xiaomin Li, Yulan Huang, Jiayong Ou, Qiujing Wei, Jieruo Gu

**Affiliations:** 1 Department of Rheumatolgy and Immunology, Huadu Affiliated Hospital of Southern Medical University, Huadu District People’s Hospital, Guangzhou, 510800 China; 2 Department of Rheumatology and Immunology, Third Affiliated Hospital of Sun Yat-sen University, 600 Tianhe Road, Guangzhou, 510630 China

**Keywords:** Gout, Hyperuricemia, High-density lipoprotein, Lipids

## Abstract

**Background:**

Dyslipidemia often concurs with hyperuricemia. Our study was to discover different lipid levels of gout and asymptomatic hyperuricemia and the predictors of sUA (serum uric acid) levels.

**Methods:**

A cross-sectional study was performed to collect demographic, clinical variables, comorbidities and laboratory testing in patients with gout and asymptomatic hyperuricemia. Group comparison was performed with Student’s *t*-test or Mann Whitney U test for continuous variables and chi-squared tests for categorical variables (Fisher’s exact test where appropriate) and to screen potential risk factors. Correlation of sUA levels with demographic and biochemical variables were performed by using correlation analysis. The variable with s *p*-value less than 0.20 during the group comparison or clinical relevance was introduced into the stepwise multiple regression model.

**Results:**

Six hundred fifty-three patients with gout and 63 patients with asymptomatic hyperuricemia (> 420 μmol/L in male and > 360 μmol/L in female) were enrolled, including 553 (84.7%) males. The mean age was 47.8 ± 16.0 years old. Elevated total cholesterol (TC) was observed in 173 (26.5%) cases with gout. Increased triglycerides (TG) and low-density lipoprotein (LDL-C) levels were observed in 242 (37.1%) cases and 270 (41.3%) cases with gout, individually. In contrast, elevated TC, TG and LDL-C levels were observed in 10 (15.9%) cases, 30 (47.6%) cases and 22 (34.9%) cases with hyperuricemia, individually. Significant differences were found in age, serum creatine, TC and erythrocyte sedimentation rate (ESR) between gout and asymptomatic hyperuricemia groups (*p* < 0.05). In patients with asymptomatic hyperuricemia, 12 (19.0%) patients had hypertension and 5 (7.9%) suffered from coronary heart diseases. Male (B = -112.7, *p* < 0.001), high-density lipoprotein (HDL-C) (B = -60.797, *p* = 0.013), body mass index (BMI) (B = 5.168, *p* = 0.024), age (B = -3.475, *p* = 0.006), age of hyperuricemia onset (B = 2.683, *p =* 0.032), and serum creatine (B = 0.534, *p* < 0.001) were predictors of sUA levels in gout patients (adjusted R^2^ = 28.7%).

**Conclusions:**

Dyslipidemia is more commonly seen in patients with gout, compared to asymptomatic hyperuricemia. HDL-C is a protective predictor of sUA levels in gout.

## Introduction

Gout is a chronic inflammatory disease characterized by recurrent joint inflammation and dysfunction caused by purine metabolism imbalance and elevated serum uric acid (sUA) whose prevalence continues to go up worldwide. As a chronic systemic inflammatory disease, gout often concurs with comorbidities such as dyslipidemia, cardiovascular disease, fatty liver disease, and renal disease [1, 2]. For example, hypertriglyceridemia is more frequently observed in gouty patients than non-gouty individuals [3]. Over the last two decades, not only in gout patients, the association of hyperuricemia with cardiovascular risk factors has also been testified following the demonstration in animal models [4] and human studies [3, 5]. Hyperuricemia has been reported to be associated with increased lipid levels [6] and decreased fibrous volume, potentially leading to raised plaque fragility [7]. Although a few studies have been conducted to investigate the association between sUA and lipid profiles in the gout population, their association in the subjects with asymptomatic hyperuricemia hasn’t been thoroughly investigated.

In consideration of the potential relationship between hyperuricemia, high lipid levels, and cardiovascular diseases, it is necessary to identify the correlation between dyslipidemia and sUA. Thus, the main purpose of this study was to discover different lipid levels of gout and asymptomatic hyperuricemia and the predictors of sUA levels in the Chinese population.

## Materials and methods

### Study population

The cross-sectional study enrolled patients consecutively admitted to the outpatient department of the Third Affiliated Hospital of Sun Yat-sen University from September 2016 to January 2019. All patients with gout fulfilled Rheumatology Association’s 2015 preliminary gout classification Criteria [8]. Asymptomatic hyperuricemia refers to the individuals who have elevated uric acid (> 420 μmol/L in male and > 360 μmol/L in female) without any joint symptom. Other inclusion criteria were listed as follows: patients should be over 16 years old. This study was approved by the ethics committee of the Third Affiliated Hospital of Sun Yat-sen University [(2017) 02–295-01] and conducted in compliance with the Helsinki Declaration. All participants gave written informed consent of the study and possible publication before enrolment.

### Data collection

All the information was collected by two trained rheumatologists during a face-to-face interview at the study visit, including a review of medical records. Demographic and clinical variables involved age, sex, body weight, height, body mass index (BMI), age of onset, disease duration of gout and hyperuricemia, clinical manifestation, past and current medication. Patient-reported questionnaires included past medical history, including cardiovascular diseases (hypertension, coronary heart disease, atherosclerosis, myocardial infarction), and other diseases.

After a 12-h fasting, a venous blood sample was obtained from each subject using the automatic biochemical analyzer 7600 (Hitachi, Ltd., Japan). Laboratory variables were also recorded such as aspartate transaminase (AST; U/L), alanine aminotransferase (ALT; U/L), total protein (TP; g/L), albumin (ALB; g/L), total bilirubin (TB; μmol/L), blood urea nitrogen (BUN; mmol/L), creatine (Cr; mmol/L), uric acid (UA; μmol/L), C-reactive protein (CRP; mg/L), erythrocyte sedimentation rate (ESR; mm in the first hour), total cholesterol (TC; mmol/L), triglyceride (TG; mmol/L), low-density lipoprotein cholesterol (LDL-C; mmol/L), and high-density lipoprotein cholesterol (HDL-C; mmol/L). Elevated TC refers to a value of more than 5.7 mmol/L. Elevated TG is defined as a value of more than 1.92 mmol/L. Elevated LDL-C means a value of more than 3.4 mmol/L. And a value of less than 0.78 denotes a decreased HDL-C value.

### Statistical analysis

Mean ± standard deviation (SD) was calculated for continuous variables and frequency and percentage for categorical variables. Group comparison was performed with Student’s *t-*test or Mann Whitney U test (Normal distribution of data was verified with the Kolmogorov-Smirnov test) for continuous variables and chi-squared tests for categorical variables (Fisher’s exact test where appropriate) and to screen potential risk factors. Correlation of sUA levels with demographic and biochemical variables including lipid levels were performed by using correlation analysis. The variable with a *p*-value of less than 0.20 during the group comparison or clinical relevance was introduced into the stepwise multiple regression model. Adjusted R^2^, standardized coefficient, and *p*-value were calculated. Statistical significance was set at two-sided *p* < 0.05. Statistical Package for Social Sciences (SPSS) software version 19 was used for all data management and analysis.

## Results

### Demographic and disease characteristics of gout patients

A total of 716 participants were recruited from September 2016 to January 2019. Demographic and disease characteristics of 653 patients with gout and 63 patients with asymptomatic hyperuricemia were shown in Table 1. Male patients (*n* = 604, 84.4%) were the majority of enrolled patients. The mean age of all the participants was 47.8 ± 16.0 years old. In patients with gout, the mean age of gout onset was 40.3 ± 15.2 years old and the mean gout duration was 8.0 ± 6.4 years. 38 (5.8%) of gout patients had tophi. The majority of male patients with gout had an age of 31–60, while female patients tended to have gout at the age of more than 40 years old. Noticeably, there were 8 female patients with gout and 3 female patients with asymptomatic hyperuricemia whose age was below 30 years old.

**Table 1:** Demographic and disease characteristics of the participants

Characteristics	All participants (*n* = 716)	Gout (*n* = 653)	Asymptomatic hyperuricemia (*n* = 63)
Male, n (%)	604 (84.4)	553 (84.7)	51 (81.0)
Age (years)	47.8 ± 16.0	48.3 ± 15.8	43.2 ± 17.9
Education level			
Primary education or below	85 (11.9)	75 (11.5)	10 (15.9)
University or above	376 (52.5)	350 (53.6)	26 (41.3)
Weight (kg)	67.5 (15.0)	63.0 (15.0)	67.0 ± 11.3
BMI (kg/ m^2^)	24.2 (5.06)	23.4 (4.7)	24.1 ± 3.2
Normal (18.5~24.9), n(%)	350 (48.9)	319 (48.9)	31 (49.2)
Overweight (25.0~29.9), n(%)	217 (30.3)	199 (30.5)	18 (28.6)
Obesity (30.0~34.9), n(%)	38 (5.3)	37 (5.7)	1 (1.6)
Severe obesity (35.0~39.9), n(%)	6 (0.8)	6 (0.9)	0
Smoking (current), n (%)	171 (23.9)	153 (23.4)	18 (28.5)
Drinking (current), n (%)	348 (48.6)	322 (49.3)	26 (41.3)
Duration of hyperuricemia (years)	6.0 (2.0–12.0)	6.0 (2.0–12.0)	6.0 (2.0–11.0)
Treatment, n (%)			
UA lowering therapy	430 (60.0)	423 (64.8)	7 (11.1)
No medication	248 (34.6)	198 (30.3)	50 (79.4)

*BMI* body mass index, *UA* uric acid, Data within normal distribution were stated as mean ± S.D., and data who did not meet normal distribution were expressed as median (interquartile range)

In patients with gout, elevated TC was observed in 173 (26.5%) cases. Increased TG and LDL-C levels were found in 242 (37.1%) cases and 270 (41.3%) cases, separately. Comparatively, elevated TC, TG, and LDL-C levels were detected in 10 (15.9%) cases, 30 (47.6%) cases and 22 (34.9%) cases with asymptomatic hyperuricemia, separately.

### Comparisons of the variables between patients with gout and patients with asymptomatic hyperuricemia

As observed in Table 2, significant differences were found in age, serum creatine, TC, and ESR between gout and asymptomatic hyperuricemia groups (*p* < 0.05). However, there was no significant difference in other variables, such as BMI, hyperuricemia duration, UA, TG, LDL-C, HDL-C, and CRP between the two groups (*p* > 0.05).

**Table 2:** Comparisons of variables between patients with gout and patients with asymptomatic hyperuricemia

Variable	Gout (*n* = 653)	Asymptomatic hyperuricemia (*n* = 63)	*p*
Demographic and clinical characteristics			
Male, n (%)	553 (84.7)	51 (81.0)	0.436 ^a^
Age (years)	48.3 ± 15.8	43.3 ± 17.9	0.018*^b^
≤30	84	20	
31–50	290	22	
51–70	214	15	
71–90	65	6	
BMI (kg/ m^2^)	24.2 (21.8–26.9)	23.9 (21.7–27.2)	0.582 ^c^
Age of hyperuricemia onset (years)	40.7 ± 15.6	36.2 ± 17.8	0.565 ^b^
Hyperuricemia duration (years)	6.0 (2.0–12.0)	6.0 (2.0–11.0)	0.935 ^c^
Hypertension, n (%)	170 (26.0)	12 (19.0)	0.224 ^a^
Coronary heart disease, n (%)	67 (10.3)	5 (7.9)	0.558 ^a^
Laboratory testing			
AST (U/L)	22.0 (17.6–27.6)	22.0 (17.2–25.8)	0.958 ^c^
ALT(U/L)	24.0 (16.0–38.6)	23.8 (15.9–32.8)	0.343 ^c^
TP(g/L)	73.9 (70.0–77.7)	74.6 (71.9–77.8)	0.519 ^c^
ALB(g/L)	44.3 (40.9–46.8)	45.5 (41.6–48.8)	0.095 ^c^
TB (μmol/L)	13.1 (9.6–17.3)	16.2 (10.9–20.0)	0.078 ^c^
BUN (mmol/L)	4.85 (4.0–6.2)	4.9 (4.1–6.5)	0.678 ^c^
Serum creatine (mmol/L)	90.0 (78.0–106.2)	83.9 (72.1–103.4)	0.036*^c^
sUA (μmol/L)	513.0 (376.5–602.0)	515.0 (438.0–592.0)	0.471 ^c^
Glucose (mmol/L)	5.23 (4.80–5.96)	5.23 (4.70–6.23)	0.959 ^c^
TC (mmol/L)	5.1 ± 1.1	4.7 ± 1.0	0.020*^b^
TG (mmol/L)	1.61 (1.12–2.45)	1.80 (1.06–2.67)	0.959 ^c^
HDL-C (mmol/L)	1.14 (0.97–1.39)	1.14 (0.99–1.36)	0.975 ^c^
LDL-C (mmol/L)	3.27 (2.62–3.87)	3.05 (2.39–3.70)	0.126 ^b^
ESR (mm/h)	31.0 (15.0–54.0)	21.0 (14.3–30.5)	0.066 ^c^
CRP (mg/L)	6.53 (4.71–25.1)	4.95 (4.50–10.3)	0.040*^c^

*BMI* body mass index, *AST* aspartate transaminase, *ALT* alanine aminotransferase, *TP* total protein, *ALB* albumin, *TB* Total bilirubin, *BUN* blood urea nitrogen, *sUA* serum uric acid, *TC* total cholesterol, *TG* triglycerides, *HDL-C* high-density lipoprotein, *LDL-C* Low-density lipoprotein, *ESR* erythrocyte sedimentation rate, *CRP* C-reactive protein; ^a^ Chi-squared test, ^b^ Student’s *t* test, ^c^ Mann Whitney U test were used for the significance of difference between two groups. * *p* < 0.05

170 (26.0%) of gout patients had hypertension compared to 12 (19.0%) patients with asymptomatic hyperuricemia while 67 (10.3%) patients with gout suffered from coronary heart diseases compared to 5 (7.9%) in the other group; however, no statistical difference was detected between the two groups in the aspect of cardiovascular diseases (*p* > 0.05).

### Correlation of serum UA levels with demographic and biochemical variables including lipid levels

Correlation between sUA levels with demographic and biochemical variables was presented in Table 3. sUA levels positively correlated with BMI, ALT, TB, BUN, serum creatine, TG, CRP in gout group and negatively correlated with age and HDL-C (*p* < 0.05). Comparatively, sUA levels showed an inverse correlation with TB, serum creatine in asymptomatic hyperuricemia (*p* < 0.05). As a whole, sUA correlated with BMI, ALT, TB, BUN, creatine, TG and CRP positively, but correlated with age and HDL-C in a negative way (*p* < 0.05).

**Table 3:** Correlation coefficients of serum uric acid levels with demographic and biochemical variables

Variables	Total		Gout		Asymptomatic hyperuricemia	
	*rho*	*p*	*rho*	*p*	*rho*	*p*
Age	−0.135*	**<0.001**	− 0.136*	**0.001**	− 0.74	0.566
Age of hyperuricemia onset	−0.091*	**0.015**	−0.088*	**0.025**	−0.126	0.326
Age of Gout onset	**–**	**–**	−0.087*	**0.027**	**–**	**–**
BMI	0.189*	**<0.001**	0.164*	**<0.001**	0.165	0.229
ALT	0.090*	**0.019**	0.086*	**0.033**	0.167	0.207
TB	0.226*	**<0.001**	0.209*	**<0.001**	0.273*	**0.046**
BUN	0.042	0.271	0.136*	**0.001**	0.071	0.589
Serum creatine	0.287*	**<0.001**	0.217*	**<0.001**	0.323*	**0.012**
Glucose	0.062	0.138	0.034	0.439	−0.028	0.837
TC	0.067	0.074	0.071	0.072	0.134	0.294
TG	0.115*	**0.002**	0.116*	**0.003**	0.113	0.376
HDL-C	−2.262*	**<0.001**	−0.282*	**<0.001**	− 0.013	0.924
LDL-C	0.013	0.734	0.011	0.778	0.071	0.692
ESR	−0.040	0.472	0.045	0.443	−0.151	0.442
CRP	0.136*	**0.008**	0.152*	**0.005**	0.138	0.474

*BMI* body mass index, *ALT* alanine aminotransferase, *TP* total protein, *TB* Total bilirubin, *BUN* blood urea nitrogen, *UA* uric acid, *TC* total cholesterol, *TG* triglycerides, *HDL-C* high-density lipoprotein, *LDL-C* Low-density lipoprotein, *ESR* erythrocyte sedimentation rate, *CRP* C-reactive protein; Spearman correlation analysis were used for the significance of difference between two groups. * *p* < 0.05

### Predictive factors for sUA levels in patients with gout

Gender, age, age of hyperuricemia onset, gout duration, BMI, ALT, creatine, TC, TG, HDL-C, LDL-C, and CRP were entered into the stepwise multiple regression model, as shown in Table 4. Male (B = -112.7, *p* < 0.001), HDL-C (B = -60.797, *p* = 0.013), BMI (B = 5.168, *p* = 0.024), age (B = -3.475, *p* = 0.006), age of hyperuricemia onset (B = 2.683, *p =* 0.032), and creatine (B = 0.534, *p* < 0.001) were predictors of sUA levels in patients with gout, whose adjusted R^2^ was equal to 28.7%.

**Table 4:** Mutiple regression analysis of risk factors for UA in patients with gout

Variables	*B*	Standardized coefficient	*t*	*p*
Gender (Male)	−112.7	−0.312	−5.378	<0.001
Age	−3.475	−0.368	−2.774	0.006
BMI	5.168	0.119	2.264	0.024
Age of hyperuricemia onset	2.683	0.284	2.156	0.032
Serum creatine	0.534	0.119	4.513	<0.001
HDL-C	−60.797	−0.145	−2.487	0.013

*BMI* body mass index, *HDL-C* high-density lipoprotein

## Discussion

In the current study, we confirmed the presence of high lipid levels including TC, TG, and LDL-C in not only patients with gout but asymptomatic hyperuricemia as well. TC was significantly higher in the gout compared to asymptomatic hyperuricemia. Our study proved a high prevalence of cardiovascular diseases as a comorbidity of gout and asymptomatic hyperuricemia. Meanwhile, serum creatine became elevated in gout patients compared with asymptomatic hyperuricemia. As cardiovascular comorbidities are frequently seen in patients with gout and play an important role in the premature mortality, patients with both gout and asymptomatic hyperuricemia should be systematically screened for cardiovascular diseases and risk factors, which should be addressed as an essential part of gout and hyperuricemia management.

UA is the end product of purine metabolism, and hyperuricemia may lead to complications such as gout, nephropathy, and urinary stones. sUA level is a biomarker for these conditions and can work as a modulator of glucose and lipid metabolism [9]. We found age was negatively correlated with sUA, possible due to the majority of the participants were male patients, which was in consistence with a recent Korean study [10]. During youth, males are at high risk of developing hyperuricemia, which may result from unhealthy lifestyle habits such as meat-based meals, alcohol intake, seafood and meat soup which are favorite food in Guangzhou. The bad habits may be corrected and result in the downtrend incidence rate with the increase of age [11]. The younger the patient had hyperuricemia or gout, the higher sUA the patients may have. However, the result was different from a previous Chinese finding [12], possibly due to the diverse gender and age structure of the participants. Our study confirms that females between 40 and 60 years old are more inclined to have gout when most females are in the perimenopause years. After menopause, hyperuricemia occurs more frequently due to estrogen deficiency [13]. Noticeably, there were 8 female patients with gout and 3 female patients with hyperuricemia whose age was below 30, indicating physicians should not simply exclude hyperuricemia by gender and age.

Except for age of onset, BMI, liver function tests including ALT and TB, and kidney function such as BUN and serum creatine correlated with sUA positively in gout patients. One of the reasons is that patients’ sUA levels become elevated mainly due to impaired renal excretion. As an inflammatory marker, CRP also correlated with sUA. It has been proposed that hyperuricemia might be partially responsible for the proinflammatory imbalance in the adipose tissue, representing an underlying mechanism of the low-grade inflammation [14]. The inflammation is among the multiple explanations of why cardiovascular diseases are often seen in gout patients. Comparatively, in patients with asymptomatic hyperuricemia, only TB and serum creatine had a positive correlation with sUA, indicating that hyperuricemia was associated with alteration of hepatobiliary metabolism [15]. Thus, TB can be added to regular examinations for individuals with asymptomatic hyperuricemia.

Hypercholesterolemia and hypertriglyceridemia remain challenging for patients with gout and hyperuricemia, resulting in an increased risk of cardiovascular and cerebrovascular disease. Hyperuricemia induced through the administration of oxonic acid causes metabolic alterations, including postprandial hypertriglyceridemia and hypertension [16]. An increased production of uric acid occurred in parallel with that of reactive oxygen species (ROS). The pro-oxidant and pro-inflammatory effects of ROS accumulation may further affect risk factors of cardiovascular diseases [17]. Besides, uric acid may contribute to endothelial dysfunction by inducing anti-proliferative effects on endothelium and impairing nitric oxide production, thus leading to inclination to cardiovascular diseases [18]. Our study confirmed the lipid profile of gout patients was typified by lower serum concentration of HDL-C, higher serum concentrations of TG, TC, and LDL-C. HDL particles are heterogeneous lipoproteins carrying a large variety of enzymes, globulins, microRNAs, complement components and acute phase reactants [19]. Diverse laboratory methods confirmed different HDL subfractions as being anti-atherogenic or associated with decreased cardiovascular risk [20]. To be noticed, patients with asymptomatic hyperuricemia could also have dyslipidemia. In our study, 47.6% of the patients had elevated TG, while 15.9% had raised TC levels. These results implied a necessity to including lipid tests into routine management of gout and even hyperuricemia.

The limitations of the current study mainly arise from the cross-sectional study design that cannot warrant a causal relationship between lipid levels and hyperuricemia. Besides, we speculate that the comorbidity rate would be underestimated due to the patient-reported method. The last but not the least, the sample size of asymptomatic hyperuricemia is limited, one of the reasons being that the patients lacking certain knowledge of asymptomatic hyperuricemia, and only seeing a doctor during a gout attack. Moreover, individuals with asymptomatic hyperuricemia are now encouraged to see a primary care provider or consult a physician online before going to a tertiary hospital. Prospective studies with a large population need to be conducted to explore the correlation between lipid levels and gout and hyperuricemia.

## Conclusions

Dyslipidemia is more commonly seen in patients with gout, compared to asymptomatic hyperuricemia. HDL-C is a protective predictor of sUA levels in gout.

## Data Availability

The datasets used and analyzed during the current study are available from the corresponding author on reasonable request.

## Abbreviations

ALB: albumin
ALT: alanine aminotransferase
AST: aspartate transaminase
BMI: body mass index
BUN: blood urea nitrogen BUN
Cr: creatine
CRP: C-reactive protein
ESR: erythrocyte sedimentation rate
HDL-C: high-density lipoprotein cholesterol
LDL-C: low-density lipoprotein cholesterol
SD: standard deviation
sUA: serum uric acid
TB: total bilirubin
TC: total cholesterol
TG: triglyceride
TP: total protein
